# Associations between plasma 24(S)-hydroxycholesterol and neuropsychological profile in fragile X syndrome

**DOI:** 10.1016/j.jlr.2025.100787

**Published:** 2025-03-27

**Authors:** Asma Laroui, Daniela Rojas, Sophie Bouhour, Mélodie Proteau-Lemieux, Luc Galarneau, Sérine Benachenhou, Armita Abolghasemi, Rosalie Plantefeve, Pierre-Luc Mallet, François Corbin, Jean-François Lepage, Artuela Çaku

**Affiliations:** 1 Department of Biochemistry and Functional Genomics, Université de Sherbrooke, Sherbrooke, Quebec, Canada; 2 Department of Psychology, University of Montreal, Montreal, Quebec, Canada; 3 Research Institute of the McGill University Health Centre, Montreal, Quebec, Canada; 4 Department of Paediatrics, Université de Sherbrooke, Sherbrooke, Quebec, Canada

**Keywords:** fragile X messenger ribonucleoprotein 1, cholesterol, oxysterols, 24(S)-hydroxycholesterol, 27-hydroxycholesterol, transcranial magnetic stimulation, clinical profile

## Abstract

Abstract Fragile X syndrome (FXS) is caused by mutations in *the fragile X mental retardation 1* gene, characterized by low plasma cholesterol levels. Considering the essential role of brain cholesterol in signaling and synaptogenesis, it is important to screen for brain cholesterol abnormalities in FXS and explore their link with neuropsychological profiles. Brain cholesterol is synthesized in situ, and the excess is primarily converted to 24(S)-hydroxycholesterol (24(S)-OHC). 27-hydroxycholesterol (27-OHC) is the major cholesterol oxidation metabolite that crosses the blood-brain barrier from peripheral circulation into the brain. Plasma levels of 24(S)-OHC and 27-OHC were quantified in FXS and control individuals. The FXS group underwent transcranial magnetic stimulation to evaluate corticospinal excitability and inhibition. The clinical profile was assessed using questionnaires evaluating specific symptoms related to autism, aberrant behaviors, and anxiety. Study results show a significant decrease in plasma levels of 24(S)-OHC in FXS as compared to controls (78.48 nM ± 20.90 vs. 99.53 nM ± 32.30; *P* = 0.006). Moreover, a negative correlation was observed between plasma levels of 24(S)-OHC and motor evoked potential (r_s_ = −0.57; *P* = 0.05) in FXS. Similarly, a negative correlation was also found between plasma levels of 24(S)-OHC and the total score of the Social Communication Questionnaire (r_s_ = −0.72; *P* = 0.002) and the Anxiety Depression and Mood Scale (r_s_ = −0.61; *P* = 0.02). The 24(S)-OHC is associated with specific neurophysiological and behavioral characteristics in individuals with FXS. Larger studies are warranted to confirm the potential of 24(S)-OHC as a reliable biomarker for FXS.

Fragile X syndrome (FXS) is the most frequent monogenetic cause of autism spectrum disorder (ASD) and inherited intellectual disability (ID) (1). FXS is caused by mutations in the *fragile X messenger ribonucleoprotein 1* gene (*FMR1*), located on chromosome X (2). As a result, there is a reduction or a total loss of its encoded protein called fragile X messenger ribonucleoprotein (FMRP)*.* FMRP is an RNA-binding protein (3) that controls the translation of many targets messenger RNAs (4) which potentially encode for proteins involved in lipid biosynthesis and catabolism (5). Metabolic anomalies have been reported in FXS, including obesity (6) and hypocholesterolemia (7, 8).

Hypocholesterolemia is defined as a decrease of plasma total cholesterol (TC) and low-density lipoprotein cholesterol (LDL-C) levels below the fifth percentile of a normalized population, adjusted for age, gender, and ethnicity (9). Berry-Kravis *et al.* (7) showed a significant decrease of plasma TC, LDL-C, and high-density lipoprotein cholesterol (HDL-C) among males with FXS compared to healthy controls. Moreover, Çaku *et al.* (8) showed that 30% of individuals with FXS meet the criteria of hypocholesterolemia. They also reported an inverse association between plasma levels of apolipoprotein B and apolipoprotein A1 with aberrant behavior and adaptive capacities, suggesting a potential alteration of cholesterol-related metabolic pathways that may affect brain function in FXS.

The brain is the dominant pool of cholesterol, containing about 25% of the body's cholesterol (10). Moreover, it produces its own cholesterol. In fact, astrocytes synthesize cholesterol and release it as cholesterol-rich apolipoprotein E lipoprotein complex into the extracellular medium. Neurons capture these lipoproteins through their LDL receptor and LDL receptor-related protein1 (11). Subsequently, apolipoprotein E undergoes recycling*,* when cholesterol levels rise above a threshold, the acyl-CoA cholesterol acyltransferase 1 removes a portion by converting it into cholesterol esters which constitute ∼1% of the TC pool in the adult brain and exist as lipid droplets (12, 13). The brain cholesterol issued to maintain cell membrane integrity and function, as well as the synthesis of myelin, which is crucial for efficient nerve signal transmission. The excess cholesterol is oxidized to 24(S)-hydroxycholesterol (24(S)-OHC) via cytochrome P450-46A1 (CYP46A1), which is almost exclusively expressed in neural cells (14). 24(S)-OHC crosses the blood-brain barrier, diffuses into the systemic circulation, and then is eliminated by the liver. This mechanism enables the primary pathway of cholesterol elimination from the brain (10, 15).

In contrast, the 27-hydroxycholesterol (27-OHC) is the most abundant peripheral derivative of cholesterol. It is ubiquitously synthesized in hepatic and extrahepatic tissues, released into the blood circulation, and transported into the brain through blood-brain barrier (16, 17, 18). There is thus a crosstalk between both oxysterols where 24(S)-OHC is the major metabolite of the brain cholesterol, while 27-OHC is the major one of the peripheral cholesterols. Brain cholesterol plays a crucial role in neural growth, synaptogenesis, biosynthesis of neurosteroids (19), and cell signaling via the modulation of the synaptic transmission (20). Depletion of cholesterol results in an alteration of different neuron receptors, specifically the N-methyl-D-aspartate receptor (NMDAR) (21). Sensory hypersensitivity (22), epileptic seizures (23, 24), and abnormal electroencephalograms (25) in FXS individuals and *FMR1* KO animal models have been reported confirming neuronal network hyperexcitability. Precisely, an imbalance between excitatory and inhibitory receptors (NMDAR) and gamma-aminobutyric acid type A (GABA_A_) has been reported in the *FMR1* KO mouse (26) and individuals with FXS (27). Transcranial magnetic stimulation (TMS) studies in FXS observed cortical hyperexcitability (28, 29) when the corticospinal tract was stimulated through the human motor cortex. Reduced plasma levels of 24(S)-OHC and 27-OHC have been reported in neurocognitive diseases including Alzheimer's (30), Huntington's (31), and Parkinson’s disease (32). All these conditions are characterized by synaptic dysfunction. Despite the evidence supporting the implication of cholesterol in synaptic function and the documented cholesterol alteration in FXS (7, 8), no study has investigated brain cholesterol metabolism in FXS. We hypothesized that individuals with FXS have an alteration in brain cholesterol metabolism that is associated with specific neurophysiological and clinical traits. In the present work, we compared plasma levels of 24(S)-OHC and 27-OHC between individuals with FXS and healthy controls and correlated them with the neurophysiological and clinical profile as evaluated by TMS and questionnaires, respectively.

## Materials and Methods

### Study population and design

**Fig. 1:**
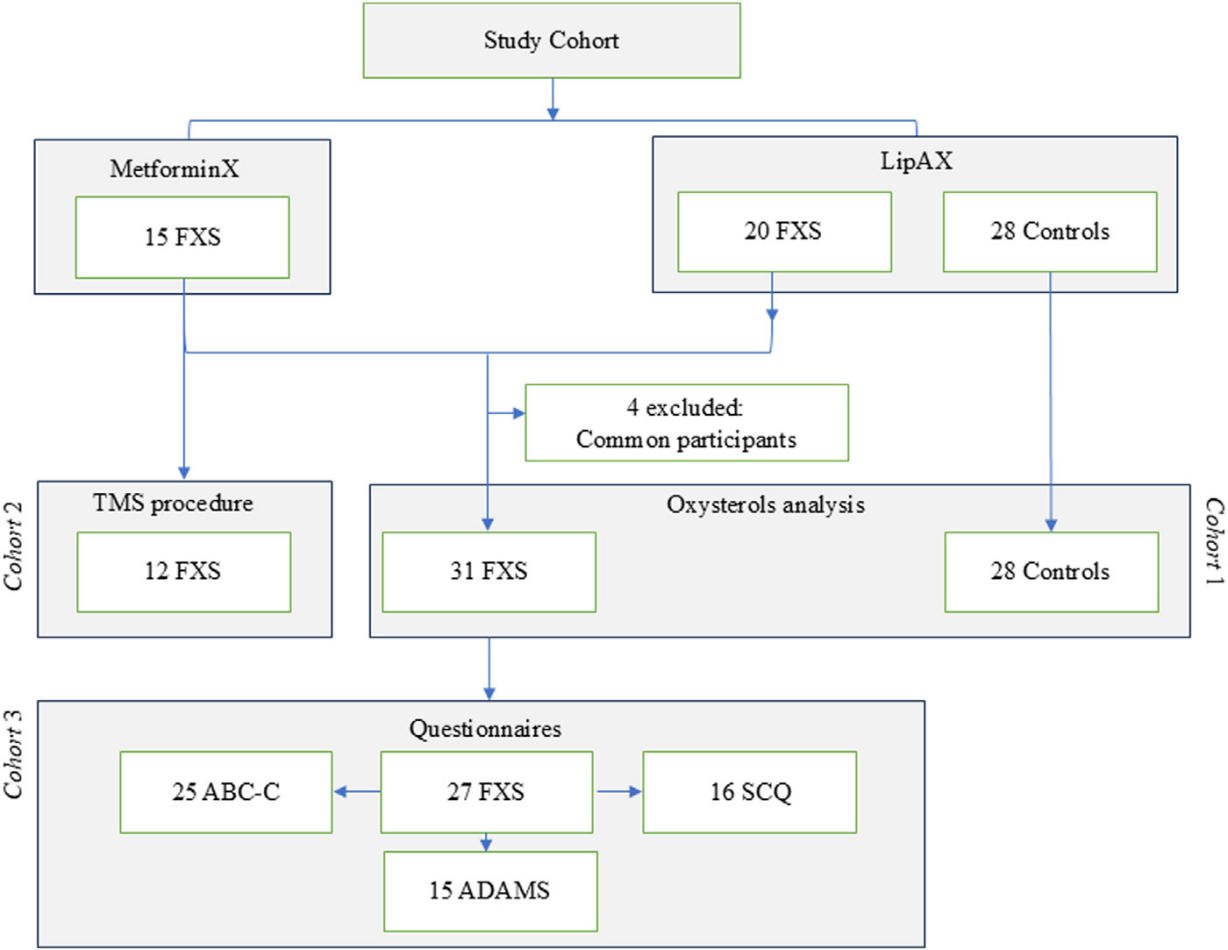
Study design.

The study population included participants from the MetforminX and LipAX cohorts, comprising a total of 31 individuals with FXS and 28 healthy controls (Fig. 1). The MetforminX trial has been previously described (33). Data obtained from pretreatment visits of 15 individuals with FXS through the MetforminX study were included in the present study. A total of 20 individuals with FXS and 28 controls were recruited through the LipAX study. Both studies were approved by the Ethics Board of the Research Center of the CIUSSS de l’Estrie-CHUS, Canada, and were performed in accordance with the Declaration of Helsinki.

Participants with FXS were recruited at the FXS clinic located at CIUSSS de l’Estrie-CHUS, while controls were recruited from the local community. The inclusion criteria consisted of males and females, aged between 12 and 45 years old, with a confirmed molecular diagnosis of FXS for the FXS group (Southern blot and polymerase chain reaction). For both FXS and control groups, the exclusion criteria were as follows: the use of medications affecting cholesterol metabolism such as hypolipidemic treatments, retinoids, corticosteroids; liver/renal dysfunction; uncontrolled hypothyroidism or hyperthyroidism; malabsorption/malnutrition; malignancy or any acute condition; diabetes; genetic dyslipidemia; and specific for controls; and any neurodevelopmental, neurocognitive, or psychiatric disorder.

There was a single study visit including a blood collection followed by an evaluation of the clinical profile in FXS participants conducted through questionnaires completed by their parents. Participants of the MetforminX cohort underwent the TMS procedure. Blood samples were collected in K2EDTA tubes (BD Vacutainer®). Plasma was recovered following successive centrifugations (300 *g* for 10 min and 2,400 *g* for 15 min to remove all cellular components) and stored at −80°C. FMRP dosage was performed on whole cell extracts from platelets (pg/10^6^ platelets) by Western blotting as previously described (34).

### Quantification of plasma lipids and oxysterols: 24(S)-OHC and 27-OHC

TC, HDL-C, and triglycerides were measured by enzymatic methods (Modular Roche P800), while LDL-C was calculated using the Friedewald formula. Apolipoprotein B, apolipoprotein A1, and lipoprotein (a) were measured by immunoturbidimetric assays (Roche Diagnostics, Cobas 501 analyzer). Measurement of 24(S)-OHC and 27-OHC was performed as previously described (35). Briefly, a volume of 200 μl of human EDTA-K2 plasma was supplemented with 20 μl of internal standards (24(R/S)-OHC-d7 and 27-OHC-d6 at 24.5 μM (10 ng/μl)). The extraction of oxysterols involved a lipid extraction step using 3 ml of DCM: MeOH (1:1) with 50 μg/ml of BHT. The alkaline hydrolysis step was performed using 10 N potassium hydroxide. Following hydrolysis, 3 ml of Dulbecco's phosphate-buffer saline was added to the extraction tube, vortexed for 30 s, and centrifuged at 2,200 *g* for 5 min. The organic layer was carefully transferred to a clean glass tube. The extracted oxysterols were then lyophilized and then reconstituted in 200 μl of MeOH containing 5 mM ammonium formate. After incubation at 30°C, the samples were transferred to HPLC autosampler vials and stored at 4°C until instrumental analysis. Oxysterol analysis was performed using ultra-performance liquid chromatography (1,290 Infinity, Agilent, Santa Clara, CA) coupled with mass spectrometry (MS) on a triple quadrupole mass spectrometer instrument equipped with an electrospray ionization source (6460 MS/MS with electrospray ionization jet stream, Agilent, Santa Clara, CA) (LC-MS/MS). The chromatographic column employed was a Luna Omega Polar C18 (2.1 × 100 mm, particle size 1.6 μm, Phenomenex, Torrance, CA). The total run time for quantifying 24(S)-OHC and 27-OHC was 17.5 min.

### TMS procedure

The TMS protocols used in the present study have been previously described in Proteau-Lemieux *et al.* (33) and Morin-Parent *et al.* (28). Measurements obtained by TMS are known to reflect specific neuronal mechanisms, as detailed in Table 1. Briefly, a figure-of-eight coil of 70 mm diameter connected to a BiStim Magstim TMS apparatus was placed tangentially to the midline (45°) on the optimal spot of the left hemisphere to induce a motor response in the contralateral first-dorsal interosseus muscle. Resulting motor contractions were recorded using electromyography with a tendon belly montage using self-adhesive circular electrodes, resulting in a motor-evoked potential (MEP) used to quantify the corticospinal excitability. Resting motor threshold (rMT) was established according to the guidelines of the International Federation of Clinical Neurophysiology (36). Test stimulus intensity for baseline MEP and paired-pulse measures were obtained at 125% of rMT. Conditioning stimulus intensity for short interval intracortical inhibition (SICI) and intracortical facilitation was set to 80% of rMT, while 125% of rMT was used for the long intracortical inhibition (LICI). The corticospinal silent period was obtained by administering a TS while participants performed an isometric contraction corresponding to 20% of the maximal force of the first-dorsal interosseus muscle as measured with a dynamic pressure gauge.

**Table 1:** TMS measurements and respective involved neuronal mechanism

TMS Measurements	Neuronal Mechanism
Resting motor threshold (rMT)	Cortical excitability mediated by Glu and NMDAR
Short interval intracortical inhibition (SICI)	Intracortical inhibition mediated by GABA_A_
Long intracortical inhibition (LICI)	Intracortical inhibition mediated by GABA_B_
Intracortical facilitation (ICF)	Cortical facilitation mediated by Glu
Cortical silent period (CSP)	Spinal and cortical inhibition mediated by GABA_A_+_B_

TMS measurements used on the participants and the neuronal mechanism corresponding for each measurement (36, 37, 38, 39).

GABA_A_, gamma-aminobutyric acid type A; GABA_B_, gamma-aminobutyric acid type B; Glu, glutamate; NMDAR, N-methyl-D-aspartate receptor; TMS, transcranial magnetic stimulation.

### Clinical evaluation

The Social Communication Questionnaire (SCQ), presented in a *YES/NO* questionnaire format, is composed of 40 items that screen symptoms associated with autism, including reciprocal social interaction, communication, repetitive behaviors, and stereotyped behaviors (37).

Social Responsiveness Scale, a 65-item questionnaire, designed to assess the severity of autism symptoms with a primary focus on social impairment, as well as common autistic behaviors such as language difficulties and stereotypy (38). The latter is composed of five domains of social awareness, social cognition, social motivation, social communication, restricted interests, and repetitive behavior, rated from 1 (not true) to 4 (almost always true) (39).

Anxiety Depression and Mood Scale (ADAMS) is a 29-item questionnaire used to assess mood disorders in persons with ID. The questionnaire evaluates five subdomains: hyperactive behavior, depressed mood, social avoidance, general anxiety, and obsessive/compulsive behavior. Each item is rated on a 4-point scale ranging from 0 (not a problem) to 3 (severe problem) (40).

Repetitive Behavior Scale-Revised is a 43-item questionnaire designed to assess the spectrum of repetitive behaviors in individuals with ASD, encompassing children, adolescents, and adults. It consists of six subscales: Stereotyped Behavior, Self-injurious Behavior, Compulsive Behavior, Routine Behavior, Sameness Behavior, and Restricted Behavior. Each item is rated on a 4-point scale: 0 (Behavior does not occur), 1 (Behavior occurs and is a mild problem), 2 (Behavior occurs and is a moderate problem), and 3 (Behavior occurs and is a severe problem) (41).

Aberrant Behavior Checklist-Community, Fragile-X version is a 58-item questionnaire used to assess aberrant behavior in individuals with ID and is specifically validated for the FXS population (42). Aberrant Behavior Checklist-Community, Fragile-X version evaluates six dimensions of behavior including irritability, hyperactivity, social unresponsiveness, social avoidance, stereotypy, and inappropriate speech. Each item is rated on a scale from 0 (not at all a problem) to 3 (severe problem) (43).

### Statistical analyses

Statistical differences between the groups were tested using student’s *t* test for quantitative variables (reported as mean ± standard deviation) and Fisher tests for categorical variables. Qualitative data were shown as a percentage. Spearman’s rank correlation was utilized to evaluate the association between oxysterols levels and the clinical profile. For all statistical tests, a *P*-value < 0.05 was considered significant. Analyses were performed using GraphPad Prism 8.

## Results

### Study population

Plasma oxysterols and plasma lipids were measured in 31 FXS and 28 healthy controls (Cohort 1) (Fig. 1). TMS results were available in a subgroup of 12 FXS participants from the MetforminX trial at the pretreatment visit (Cohort 2) (Fig. 1). Clinical data including score results of questionnaires were obtained from 27 FXS participants of MetforminX and LipaX studies (Cohort 3) (Fig. 1). The characteristics of each cohort are shown in Table 2. A total of 31 participants with FXS and 28 healthy controls were recruited, including 84% and 75% males, respectively. The median age of FXS and control groups as well as male-FXS and full mutation subgroups were comparable (supplemental Table S1). The BMI ranged from 16.3 to 43 (kg/m^2^) for the participants with FXS and from 17.3 to 37 (kg/m^2^) for the controls. The age range and BMIs were comparable with no statistical differences between cohorts. 74% of the FXS group had a full mutation diagnosis. The majority of FXS individuals with detectable FMRP were mosaics (4.6–42.9 pg/10^6^ platelets). Hypocholesterolemia was observed in 36% of participants with FXS. Half of the FXS group was medicated: 26% were taking antipsychotics and/or antidepressants, while the majority (48%) were on other types of medications such as vitamins, contraceptives, and thyroid medication.

**Table 2:** Characteristics of the study cohorts

Cohorts	Controls	1-Oxysterol	2-TMS	3-Questionnaires
		FXS	FXS	FXS
N	28	31	12	27
Males N (%)^a^	21 (75)	26 (84)	10 (83)	22 (81)
Age, median (range)^b^	26.50 (13–42)	28 (12–44)	28 (17–44)	28 (14–44)
BMI median (range)^b^	26.5 (17.3–37)	26.4 (16.3–43)	29.37 (19.30–42.27)	26.30 (16.30–42.95)
Type of mutation N (%)				
Full mutation	-	23 (74)	9 (75)	21 (77.78)
Mosaicism	-	8 (26)	3 (25)	6 (22.22)
FMRP (pg/10^6^) N (%)				
Not detectable	-	16 (52)	6 (50)	15 (56)
Detectable	-	11 (35)	6 (50)	10 (37)
Not measured	-	4 (13)	-	2 (7)
Lipid profile				
˂5^th percentile^ N (%)				
TC	1 (4)	11 (36)	3 (25)	10 (37)
HDL-C	1 (4)	4 (13)	2 (17)	2 (7)
LDL-C	3 (11)	9 (29)	2 (17)	8 (30)
Triglycerides	1 (4)	2 (6.4)	1 (8)	1 (4)
Medication				
Medicated N (%)	8 (29)	16 (52)	5 (42)	15 (56)
Antipsychotic	0	8 (26)	2 (17)	8 (30)
Antidepressant	1 (4)	8 (26)	3 (25)	8 (30)
Stimulant	0	2 (6)	0	2 (7)
Other	7 (25)	15 (48)	8 (67)	12 (44)
Not medicated N (%)	20 (71)	15 (48)	7 (58)	12 (44)

FMRP, fragile X messenger ribonucleoprotein; FXS, Fragile X syndrome; HDL-C, high-density lipoprotein cholesterol; LDL-C, low-density lipoprotein cholesterol; TMS, transcranial magnetic stimulation.

a Fisher exact test.

b Mann-Whitney test.

### Oxysterol levels in individuals with FXS and controls

**Fig. 2:**
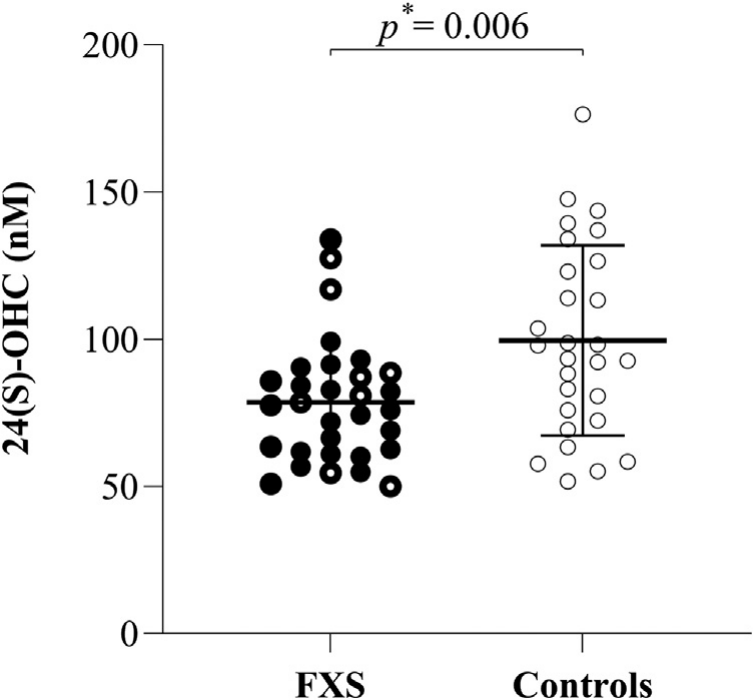
Plasma levels of total 24(S)OHC, quantified following saponification, in individuals with FXS (n = 31) and healthy controls (n = 28).  Individuals with FXS,  Individuals with FXS carrying full mutation,  Controls, ∗ Mann-Whitney test. The bar represents the mean, and the whiskers represent the standard deviation.

Plasma levels of 24(S)-OHC were significantly lower in the FXS group compared to the control group (78.48 nM ± 20.90 vs. 99.53 ± 32.30 nM; *P* = 0.006), as shown in Fig. 2. The subgroup of individuals with FXS carrying full mutation were even lower as compared to controls (76.03 nM ± 18.48 vs. 99.53 nM ± 32.30; *P* = 0.005). Similar results were obtained when comparing males only (74.80 nM ± 18.74 vs. 91.98 nM ± 28.66; *P* = 0.027) (supplemental Table S2). Plasma levels of 27-OHC were also lower in the FXS group compared to the control group, although the difference did not reach statistical significance (254.5 nM ± 77.01 vs. 282.2 nM ± 116.3; *P* = 0.693) (supplemental Table S2).

Plasma levels of 24(S)-OHC correlated positively with plasma TC (r_s_ = 0.66; *p* ˂ 0.0001) and LDL-C (r_s_ = 0.45; *P* = 0.01). Comparable results were obtained for plasma 27-OHC when correlated with TC (r_s_ = 0.43; *P* = 0.01) and LDL-C (r_s_ = 0.42; *P* = 0.02). No association was found between BMI and plasma levels of 24(S)-OHC and 27-OHC.

### Association of plasma oxysterols with TMS measurements

**Fig. 3:**
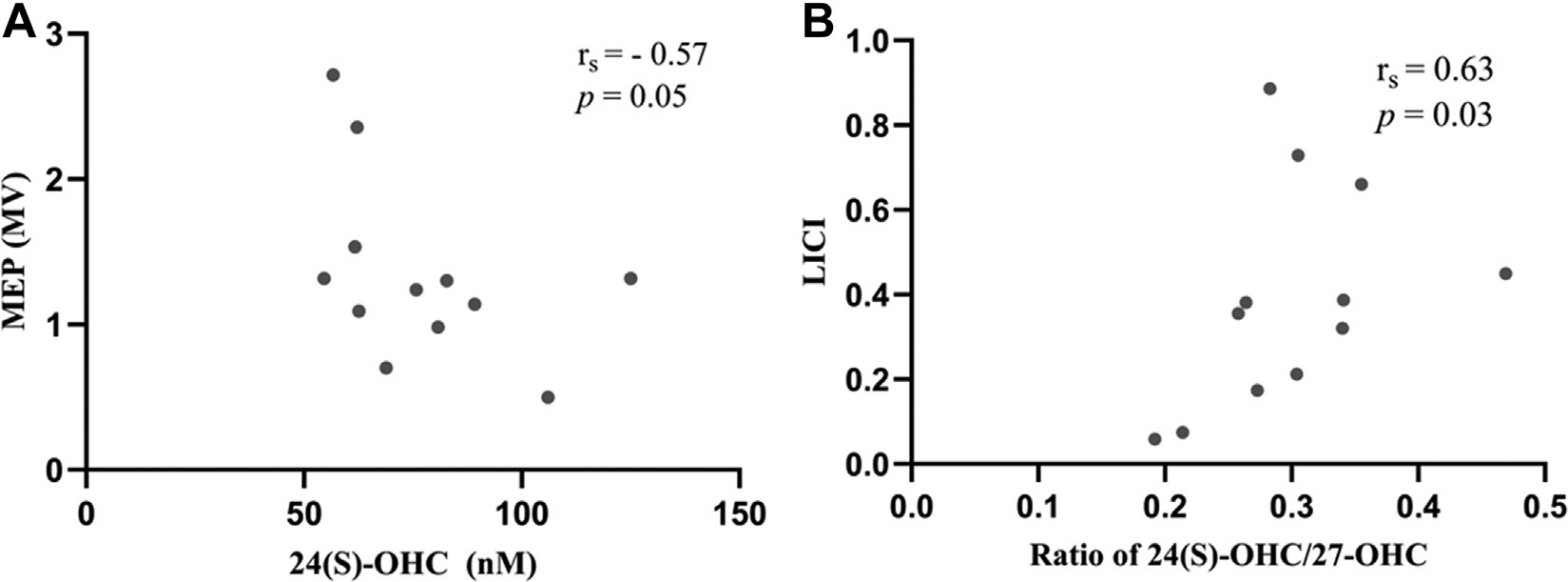
Correlation between 24(S)-OHC and ratio of 24(S)-OHC/27-OHC and TMS measurements in individuals with FXS (n = 12). (A) Correlation between 24(S)-OHC and MEP; (B) correlation between ratio of 24(S)-OHC/27-OHC and LICI. MV, millivolts.

Spearman correlation conducted between TMS measurements and oxysterols in 12 individuals with FXS revealed a negative association between plasma levels of 24(S)-OHC and 27-OHC with the MEP (r_s_ = −0.57, *P* = 0.05; r_s_ = −0.68, and *P* = 0.018, respectively), as shown in Fig. 3A. A significant positive correlation was obtained between ratio of 24(S)-OHC/27-OHC and LICI (r_s_ = 0.63, *P* = 0.03) (Fig. 3B). Correlation results between oxysterols and TMS variables in FXS group and controls are shown in supplemental Tables S3 and S4, respectively.

### Association of plasma oxysterols with clinical profile in FXS

**Fig. 4:**
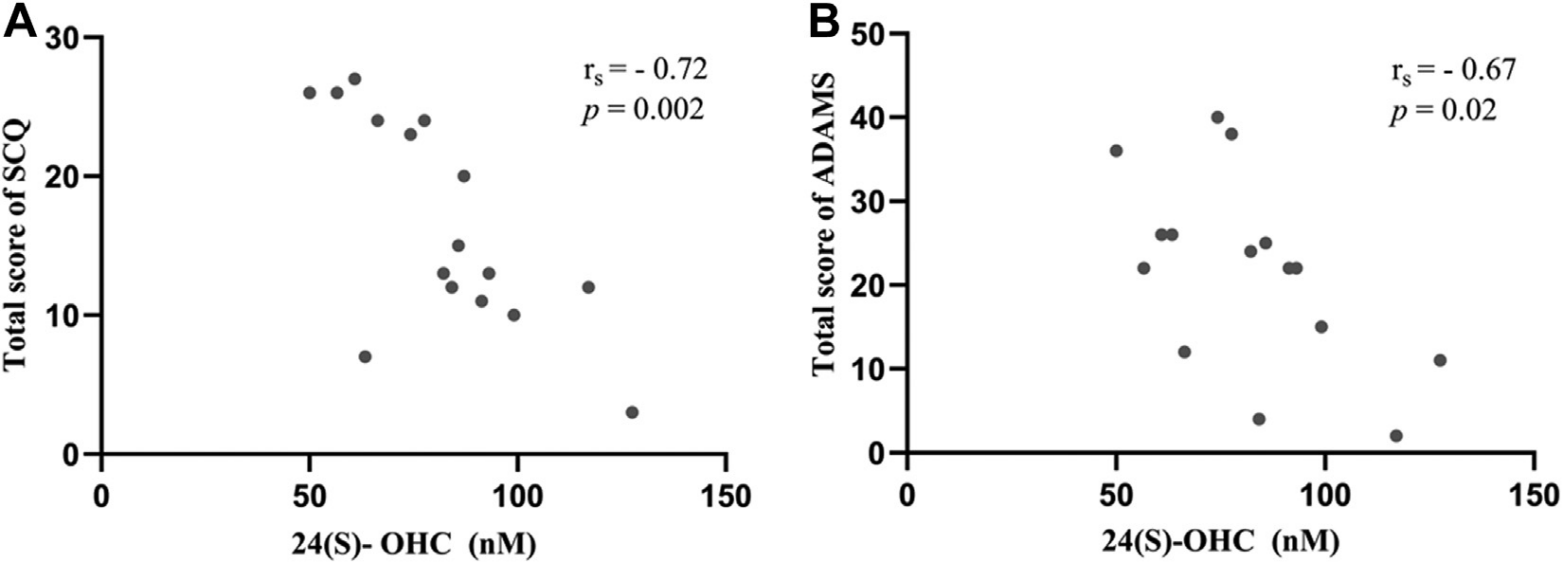
Correlation between 24(S)-OHC and clinical profile in individuals with FXS. (A) Negative correlation between plasma levels of 24(S)-OHC and the total score of SCQ (n = 16). (B) Negative correlation between 24(S)-OHC and total score of ADAMS (n = 15).

There was a statistically significant negative association between plasma levels of 24(S)-OHC and the total scores of SCQ (r_s_ = −0.72, *P* = 0.002) and ADAMS (r_s_ = −0.67, *P* = 0.02), in individuals with FXS as shown in Fig. 4.

## Discussion

This study remains the first to investigate brain cholesterol in humans with FXS. Overall, our results show that the major metabolites of brain and peripheral cholesterol, 24(S)-OHC and 27-OHC, respectively are decreased in FXS. Furthermore, 24(S)-OHC plasma levels are associated with specific traits of neurophysiological and clinical profile in this population.

The plasma levels of 24(S)-OHC were significantly reduced in the FXS individuals, with an increased difference observed in FXS carrying full mutation. Similar results were previously reported in other neurodegenerative diseases such as Alzheimer’s disease (30), multiple sclerosis (44), and Huntington’s disease (45). In FXS, studies on the brain cholesterol remain limited. Specifically, Parente *et al*. (46) reported an increase of brain cholesterol content levels in the amygdala of a Fmr1-Δexon 8 rat model of FXS. A significant decrease of CYP46A1, the enzyme that metabolizes brain cholesterol into 24(S)-OHC, was also observed along with a decrease of the sterol regulatory binding protein 2. The latter is responsible for maintaining brain cholesterol homeostasis by controlling the transcriptional machinery of genes involved in cholesterol synthesis depending on cholesterol cellular levels (14). Moreover, Talvio *et al*. (47) reported a decrease of ATP-binding cassette transporter A1 (ABCA1) (cholesterol membrane transporter to the extracellular environment) in both human and mouse iPSC-derived astrocyte model of FXS. This alteration was associated with an accumulation of cholesterol and its precursors in FXS mouse astrocytes. ABCA1 expression is primarily regulated by LXR activity, which is influenced by cholesterol levels (48). However, when cholesterol levels are elevated, increased production of oxysterols activates LXRs and leads to enhanced ABCA1 expression (49). These observations suggest a potential decrease of the CYP46A1 enzyme activity in FXS that might explain a reduction of plasma 24(S)-OHC observed in our study. Our results are consistent with these two reports. These results altogether suggest a decrease in brain cholesterol degradation into 24(S)-OHC and a consecutive increase of cholesterol content. The latter leads to a decrease of cleaved sterol regulatory binding protein 2 and ABCA1 as a result of the classical homeostatic response to intracellular cholesterol accumulation (46). Alternatively, Ren *et al.* (50) observed a reduction in enzymes involved in cholesterol biosynthesis pathway (Cyp51A1 and Sc4mol (MSM01)), as well as a decrease of cholesterol content in astrocytes from human FXS-derived induced pluripotent stem cells (hiPSCs). The authors reported that human FXS-derived induced pluripotent stem cell–derived astrocytes shared more similarities with fetal astrocytes than with mature human astrocytes, which may explain the immaturity in expression of cholesterol biosynthetic enzymes. We should note that the regulation of cholesterol synthesis and degradation is complex, and the underlying cause of the alteration of brain cholesterol metabolism in FXS remains unclear. However, considering the further reduction of 24(S)-OHC in FXS individuals with full mutation as well as the role of FMRP in the regulation of expression of numerous brain proteins (51), we hypothesize that FMRP might control the expression and/or activity of CYP46A1. Further, in vitro studies are warranted to explore the underlying mechanism and validate this hypothesis.

Several factors might influence plasma levels of 24(S)-OHC including, age and sex. There is an age-dependent variation of plasma levels of 24(S)-OHC (52, 53). The highest levels are reported in the first and second year of life and then decline to reach adult levels around the age of 12–15 years (52). In the current study, cohorts were limited to the individuals aged 12 years and older. Since only two participants were under 15 years old, the potential effect of age as a variable affecting plasma levels of 24(S)-OHC was minimal. Furthermore, we conducted a correlation analysis to assess age-related variation in plasma levels of 24(S)-OHC. Our result showed no significant association in either FXS or control group. In addition, sex has a specific effect on cholesterol metabolism (54, 55): females display higher levels of 24(S)-OHC as compared to males, both in healthy controls (55) or individuals with mental disorder (55). In accordance with previous reports, we observed higher plasma levels of 24(S)-OHC in female controls as compared to male controls. However, considering the limited number of females, we could not conclude about sex-based differences of plasma 24(S)-OHC. However, the number of males and females in FXS and control groups was not statistically different to potentially influence the levels of 24(S)-OHC.

This study represents a first attempt to screen the potential association between brain cholesterol levels and neurophysiologic profile of individuals with FXS. An inverse relationship was observed between plasma 24(S)-OHC and MEP: individuals with lower plasma 24(S)-OHC displayed higher corticospinal excitability. An increase in MEP amplitude obtained at comparable intensities can be interpreted as a sign of heightened excitability and/or conductivity of cortical neurons (56). Furthermore, a positive, statistically significant association was observed between the ratio of 24(S)-OHC/27-OHC and LICI measurement. LICI describes the suppression of neuronal activity following double-pulse TMS and reflects intracortical inhibition mediated by the GABAergic system, specifically through postsynaptic GABA_B_ receptors (57). Taken together, these results suggest that individuals with FXS displaying lower plasma levels of 24(S)-OHC showed higher cortical excitability and lower GABA_B_-mediated intracortical inhibition compared to those with higher cholesterol levels. Our findings align with previous studies that have reported an imbalance between cortical excitation and inhibition in FXS resulting in cortical hyperexcitability. For instance, we previously reported a reduction of GABA_A_-mediated inhibition and an increase of GABA_B_-mediated inhibition, as shown respectively by an increase of SICI and a decrease of LICI in individuals with FXS as compared to healthy controls (28).

In the present study, no significant association between oxysterols and GABA_A_-mediated inhibition was observed, suggesting that in FXS, brain cholesterol alterations may primarily impact GABA_B_-mediated inhibition. The link between brain cholesterol and the GABA_A_ receptors has been reported. Both, cholesterol depletion and enrichment can affect their function, suggesting that an optimal level of brain cholesterol is essential for optimal receptor activity (58). Although, there is limited research about the link between cholesterol and GABA_A_ receptors, similar mechanisms might apply to GABA_B_ receptors. The present results suggest a link between brain cholesterol degradation and GABA_B_ receptors; however, further research is warranted to establish this relationship.

Our results revealed a significant negative association between plasma levels of 24(S)-OHC and the total score of SCQ and ADAMS in FXS, indicating a link between brain cholesterol catabolism and autistic behaviors and anxiety, respectively. A similar relationship between SCQ and cholesterol content in lipid rafts isolated from platelets was reported in individuals with FXS (59). We should note that ASD is a common feature in FXS, with 30%–54% of FXS males meeting diagnostic criteria for ASD (60). Hypocholesterolemia has been reported in 23% of individuals with ASD (61); however, only one study investigated brain cholesterol. Specifically, Graya et *al*. (62) found an increase in plasma levels of 24(S)-OHC in 36 children with ASD as compared to 38 healthy controls matched for age and sex. Considering the limited research on brain cholesterol in individuals affected with ASD and/or FXS, other studies including the measurement of other brain-derived oxysterols are important to further understand alterations of brain cholesterol in living subjects. Anxiety is also a common condition observed in many neurodevelopmental and mental disorders. Up to 86% of males and 77% of females with FXS meet diagnostic criteria of anxiety disorder (40). To our knowledge, this is a first report in FXS showing an association between brain cholesterol catabolism and anxiety. Nevertheless, Guidara *et al*. (63) have shown a significant decrease of 24(S)-OHC plasma levels in patients with bipolar disorder, the underlying mechanism remains unknown.

The results of the present study should be interpreted in the light of some limitations. First, levels of brain cholesterol metabolites were quantified using peripheral blood samples rather than cerebrospinal fluid. This approach may not accurately reflect the state of central lipids. Second, the number of participants was limited to conclude about alterations of brain cholesterol metabolite in FXS. Although the present findings suggest the presence of such perturbations, further studies including a larger sample size as well as the measurement of other brain-derived oxysterols are needed to explore and potentially understand the metabolism of brain cholesterol in living subjects. Third, this study did not include magnetic resonance imaging data, which could have provided quantitative measurements of GABA neurotransmitter levels in the brain and helped to better understand the correlation between brain cholesterol metabolites and these specific neurochemical profiles.

In conclusion, this study represents a significant advancement in our understanding of FXS, providing the first evidence of reduced brain cholesterol catabolism in individuals with this condition. Our findings demonstrate a novel association between plasma levels of 24(S)-OHC, a brain-specific cholesterol metabolite, and key neurophysiological and clinical features of FXS. Moreover, our results identify 24(S)-OHC as a promising biomarker candidate to predict distinct neurophysiological and clinical profiles in FXS. Future studies should aim to replicate these findings in larger cohorts, investigate the mechanistic links between cholesterol metabolism and FXS symptoms, and explore the potential of cholesterol-targeted therapies in FXS treatment.

## Data availability

The fundamental data of this study are included in the article. However, additional datasets (raw data) are available from the corresponding author on reasonable request.

## Supplemental data

This article contains supplemental data.

## Conflict of interest

The authors declare that they have no conflicts of interest with the contents of this article.

## Supplemental data

Suplementary data
