# Supplementary material for: Associations between plasma 24(S)-hydroxycholesterol and neuropsychological profile in fragile X syndrome
Source: J Lipid Res. 2025 Mar 27;66(5):100787. doi: 10.1016/j.jlr.2025.100787 (PMC12088753; doi:10.1016/j.jlr.2025.100787)
Supplement: Suplementary data [file mmc1.docx]

**Supplementary table 1.** Characteristics of the study cohorts.

| **Cohort** | **FXS full mutation** | **Males group** | |
| --- | --- | --- | --- |
| **Group** |  | Controls | FXS |
| **All (N)** | 23 | 21 | 26 |
| Males N (%) * | 22 (96) | 21 (75) | 26 (84) |
| Age, median (range)** | 28 (14 - 43) | 27.50 (13 - 39) | 27.50 (12 - 43) |
| BMI median (range)** | 26.45 (17.8 - 43) | 26.10 (17.30 -36) | 25.19 (17.8 - 43) |
| **Type of Mutation** N (%) |  |  |  |
| Full mutation | 23 (74) | - | 22 (85) |
| Mosaicism | 8 (26) | - | 4 (15) |
| **FMRP (pg/10^6^)** N (%) |  |  |  |
| Not detectable | 15 (65) | - | 16 (62) |
| Detectable | 6 (26) | - | 6 (23) |
| Not measured | 2 (9) | - | 4 (15) |
| **Lipide profile** |  |  |  |
| ˂ 5^e percentile^ N (%) |  |  |  |
| TC | 8 (35) | 1 (5) | 11 (42) |
| HDL-C | 3 (13) | 1 (5) | 4 (15) |
| LDL-C | 6 (26) | 3 (14) | 9 (35) |
| Triglycerides | 1 (4) | 1 (5) | 2 (8) |
| **Medicated N** (%) | 10 | 0 | 12 |
| **No medicated N** (%) | 13 | 0 | 14 |

^*^Fisher exact test.

^**^Mann-Whitney test.

**Supplementary Table 2.** Oxysterols plasma levels in individuals with FXS and controls.

|  | ***Oxysterols*** | | |
| --- | --- | --- | --- |
| ***Group*** | 24(S)-OHC | 27-OHC | 24(S)-OHC/27-OHC |
| All controls (*n*=28) | 99.53 nM ± 32.30 | 282.2 nM ± 116.3 | 0.373 ± 0.173 |
| All FXS (*n*= 31) | 78.48 nM ± 20.90 | 254.5 nM ± 77.01 (*n*=30) | 0.335 ± 0.120 (*n*=30) |
| *p-value* * | 0.006 | 0.693 | 0.305 |
| All controls (*n*=28) | 99.53 nM ± 32.30 | 282.2 nM ± 116.3 | 0.373 ± 0.173 |
| FXS with full mutation (*n*=23) | 76.03 nM ± 18.48 | 260.1 nM ± 78.02 | 0.308 ± 0.078 |
| *p-value* * | 0.005 | 0.901 | 0.135 |
| Male controls (*n*=21) | 91.98 nM ± 28.66 | 295 nM ± 128.4 (*n*=19) | 0.3148 nM ± 0.09 (*n*=19) |
| Males with FXS (*n*= 26) | 74.80 nM ± 18.74 | 255.2 nM ± 82.20 (*n*=25) | 0.3180 nM ± 0.096 (*n*=25) |
| *p-value* * | 0.027 | 0.485 |  |

*Mann-Whitney test.

**Supplementary Table 3.** Association of plasma levels of 24(S)-OHC, 27-OHC, and ratio of 24(S)-OHC/27-OHC with TMS measurements in individuals with FXS.

|  | *24(S)-OHC* | | *27-OHC* | | *24(S)-/27-OHC* | |
| --- | --- | --- | --- | --- | --- | --- |
| N= 12 | *r*_s_ | *p* | *r*_s_ | *p* | *r*_s_ | *p* |
| TMS variables |  |  |  |  |  |  |
| rMT | 0.14 | 0.655 | 0.35 | 0.262 | -0.04 | 0.899 |
| Intensity (1Mv) | 0.02 | 0.960 | 0.31 | 0.32 | -0.11 | 0.727 |
| MEP baseline | -0.57 | **0.05** | -0.68 | **0.018** | -0.01 | 0.974 |
| SICI (2 ms) | -0.29 | 0.366 | -0.50 | 0.104 | 0.27 | 0.391 |
| SICI (4 ms) | -0.31 | 0.319 | -0.16 | 0.619 | 0.01 | 0.974 |
| ICF (10 ms) | -0.39 | 0.21 | -0.45 | 0.147 | 0.29 | 0.354 |
| ICF (15 ms) | -0.14 | 0.667 | -0.14 | 0.667 | 0.22 | 0.499 |
| SICF (3 ms) | -0.23 | 0.471 | -0.11 | 0.733 | -0.06 | 0.851 |
| LICI (100 ms) | 0.14 | 0.667 | 0.03 | 0.939 | -0.08 | 0.800 |
| LICI (200 ms) | 0.29 | 0.366 | -0.07 | 0.835 | 0.63 | **0.032** |
| CSP | 0.09 | 0.811 | -0.03 | 0.946 | 0.38 | 0.279 |

Significant r_s_ values (*p* ˂ 0.05) are given in bold.

**Supplementary Table 4.** Association of plasma levels of 24(S)-OHC, 27-OHC, and ratio of 24(S)OHC-/27-OHC with TMS measurements in healthy controls.

|  | *24(S)-OHC* | | *27-OHC* | | *24(S)-/27-OHC* | |
| --- | --- | --- | --- | --- | --- | --- |
| N= 9 | *r*_s_ | *p* | *r*_s_ | *p* | *r*_s_ | *p* |
| TMS variables |  |  |  |  |  |  |
| rMT | -0.03 | 0.945 | 0.46 | 0.222 | -0.08 | 0,843 |
| Intensity (1Mv) | 0.02 | 0.958 | 0.41 | 0.273 | -0.008 | 0.992 |
| MEP baseline | 0.43 | 0.25 | 0.18 | 0.644 | -0.05 | 0.912 |
| SICI (2 ms) | 0.53 | 0.147 | 0.17 | 0.678 | 0.82 | **0.011** |
| SICI (4 ms) | 0.60 | 0.097 | 0.48 | 0.194 | 0.45 | 0.23 |
| ICF (10 ms) | -0.05 | 0.912 | 0.07 | 0,880 | -0.13 | 0.743 |
| ICF (15 ms) | 0.00 | >0.99 | 0.22 | 0.581 | 0.000 | >0.99 |
| SICF (3 ms) | -0.33 | 0.385 | 0.20 | 0.613 | -0.45 | 0.23 |
| LICI (100 ms) | 0.08 | 0.843 | 0.45 | -0.230 | 0.23 | 0.552 |
| LICI (200 ms) | 0.30 | 0.437 | -0.08 | 0.843 | 0.05 | 0.912 |
| CSP | -0.23 | 0.552 | -0.03 | 0.946 | -0.62 | 0.086 |
